# A North American *Yersinia pestis* Draft Genome Sequence: SNPs and Phylogenetic Analysis

**DOI:** 10.1371/journal.pone.0000220

**Published:** 2007-02-21

**Authors:** Jeffrey W. Touchman, David M. Wagner, Jicheng Hao, Stephen D. Mastrian, Maulik K. Shah, Amy J. Vogler, Christopher J. Allender, Erin A. Clark, Debbie S. Benitez, David J. Youngkin, Jessica M. Girard, Raymond K. Auerbach, Stephen M. Beckstrom-Sternberg, Paul Keim

**Affiliations:** 1 The Translational Genomics Research Institute, Phoenix, Ariziona, United States of America; 2 The Biodesign Institute, Arizona State University, Tempe, Ariziona, United States of America; 3 Center for Microbial Genetics and Genomics, Northern Arizona University, Flagstaff, Ariziona, United States of America; Institute of Human Virology, United States of America

## Abstract

**Background:**

*Yersinia pestis*, the causative agent of plague, is responsible for some of the greatest epidemic scourges of mankind. It is widespread in the western United States, although it has only been present there for just over 100 years. As a result, there has been very little time for diversity to accumulate in this region. Much of the diversity that has been detected among North American isolates is at loci that mutate too quickly to accurately reconstruct large-scale phylogenetic patterns. Slowly-evolving but stable markers such as SNPs could be useful for this purpose, but are difficult to identify due to the monomorphic nature of North American isolates.

**Methodology/Principal Findings:**

To identify SNPs that are polymorphic among North American populations of *Y. pestis*, a gapped genome sequence of *Y. pestis* strain FV-1 was generated. Sequence comparison of FV-1 with another North American strain, CO92, identified 19 new SNP loci that differ among North American isolates.

**Conclusions/Significance:**

The 19 SNP loci identified in this study should facilitate additional studies of the genetic population structure of *Y. pestis* across North America.

## Introduction

Plague is a disease of both historical and current importance to global health. The first accounts of this disease are from Europe in the 6th century during the Justinian rule of the Roman Empire. After a century of epidemics, plague disappeared until the Middle Ages (14th century), when the devastation of the epidemic known as the Black Death killed millions of Europeans. That second pandemic raged until the 17th century when plague again disappeared. The third and current pandemic began in the mid 19th century when the disease emerged from eastern China. At that time, worldwide commerce allowed it to spread to all inhabited continents. As a result, plague became ecologically established in many locations, including western North America, and active foci are currently found on all continents except Australia and Antarctica [1]. In the 20th century, plague was developed as a biological weapon by Japan, the United States, and the former Soviet Union [2]. It is currently listed as a Category A biological agent for public health preparedness, due to its potential use in biological terrorism [3].

Plague is caused by the Gram negative bacterium *Yersinia pestis*. Genetic analyses suggest that *Y. pestis* is a recently emerged clone that evolved from *Y. pseudotuberculosis* within the last 20,000 years [4]–[6]. Despite this close genetic relationship, *Y. pestis* and *Y. pseudotuberculosis* exhibit radically different life cycles: *Y. pseudotuberculosis* lives directly in the environment, whereas *Y. pestis* is an obligate pathogen that is found only in arthropod vectors or mammalian hosts. This dual life must be evolutionarily challenging and represents a very different and perhaps more restricted niche compared to that of *Y. pseudotuberculosis*. Comparisons of complete genome sequences from strains of these two species support the idea of a more restricted niche for *Y. pestis*, as up to 13% of the *Y. pseudotuberculosis* genes are no longer active in *Y. pestis* [7]. These same analyses identified 32 chromosomal genes unique to *Y. pestis* [7]. These new genes, together with the two plasmids that are unique to *Y. pestis*, may have facilitated the radical and rapid niche shift observed in this recently emerged pathogen.

Plague was introduced to North America quite recently, within the last 125 years. In the late 1890s and early 1900s, the disease was first documented in human and non-native rat populations in several coastal cities in North America, including: Galveston, Los Angeles, San Francisco, and Seattle [8]. Following improved hygiene and other measures that decreased populations of non-native rats, the plague mostly disappeared from these cities. However, in 1908, it was documented as the causative agent of large-scale epizootics in native rodent populations near San Francisco [8]. It is commonly accepted that, following this initial introduction into the native fauna, plague spread rapidly through rodent populations, reaching its current North American distribution by 1950. That distribution is limited to the 17 western-most states, with the eastern boundary consisting roughly of 100°W longitude [9]. Plague is now endemic in the western USA, with major foci in California and the Four Corners region, which includes portions of Arizona, Colorado, New Mexico, and Utah. In the United States, the disease is usually cryptic but occasionally responds to environmental cues [10], [11], which results in large-scale epizootics among susceptible hosts [12]. These epizootics are primarily associated with two closely-related groups of rodents: prairie dogs (*Cynomys* spp.) and ground squirrels (*Spermophilus* spp.).

Molecular analyses used to differentiate and characterize strains from regional populations across North America are hampered by a lack of informative genetic markers. Variable number tandem repeat markers provide good resolution at a regional scale [12] but are less useful at defining the major phylogenetic patterns across North America (Wagner et al., unpublished). To explore genomic variation in North American plague, we have sequenced a second North American isolate, FV-1, from a recent epizootic [12] and characterized the differences between it and the available sequence of another North American strain, CO92 [13], which was isolated in Colorado. We report here the gapped genomic sequencing of strain FV-1, the comparative analysis of FV-1 with CO92 that has resulted in the identification of multiple informative single nucleotide polymorphism (SNP) markers, and the phylogenetic stratification of 22 different North American *Y. pestis* isolates using these markers.

## Results and Discussion

### Gapped-genome sequencing of *Yersinia pestis* strain FV-1

The *Y. pestis* FV-1 isolate used in this study was obtained from a plague epizootic in a Gunnison's prairie dog (*Cynomys gunnisoni*) colony near the city of Flagstaff, Arizona, U.S.A. in 2001 [12]. The genome of strain FV-1 was sequenced by a whole-genome shotgun strategy that achieved 11-fold average sequence coverage per base pair. A total of 62,649 random sequence reads were assembled into 444 contiguous segments with an N50 length (the length X such that 50% of the sequence is contained in contigs of length X or greater) of 19,182 base pairs. We note that the number of contigs is somewhat high given the level of sequence coverage achieved. We attribute this to the repeat-rich nature of the plague genome (3.7%, [13]) that can confound assembly programs. Nevertheless, we estimate that roughly 94.4% of the genome sequence of FV-1 is contained in this dataset by aligning it to the highly similar strain CO92. Due to the high level of sequence coverage, the quality of the resulting sequence data is high, with 98% percent of consensus base pairs possessing an estimated error rate of less than 1 in 1,000. The program Arachne [14] was used to assemble the genome and no assembly errors were observed based on the concordance of forward and reverse read-pairs.

### Single Nucleotide Polymorphisms

The gapped genome sequence of *Y. pestis* strain FV-1 was aligned to the complete genome sequence of strain CO92 (GenBank no. NC_003143) using the program MUMmer [15]. We discovered 32 putative single nucleotide polymorphisms (SNPs) by extracting single-base-pair mismatches from the alignment of the two genomes. A criteria was enforced that required that the SNP nucleotide in the FV-1 sequence be of high quality (see Methods). We further required the presence of 250 base pairs on either side of the putative SNP in FV-1 to avoid potential poor quality SNPs at contig ends. SNPs were verified by testing genomic DNA from both discovery strains for the allelic difference at each putative SNP locus. Out of 32 putative SNPs identified through genome sequence comparison, 19 were confirmed in both the FV-1 and CO92 genomes by direct testing of purified genomic DNA. Of note, 11 of the 32 SNPs calls were confirmed in FV-1 DNA but were incorrect in CO92 DNA, suggesting that perhaps our CO92 isolate used for SNP validation is slightly dissimilar to the strain sequenced by Parkhill et al. [13], or the publicly available sequence of strain CO92 contains an error at these 11 particular positions. An error rate of <3×10−6 would be excellent if the latter is the case.

The 19 confirmed SNPs were located within both coding and non-coding regions of the genome; 14 are located within genes, the other 5 in intergenic regions (Table 1). Based on the CO92 gene density of 83.9% [13], this ratio reflects a modest bias towards a lower occurrence of SNPs within genes (binomial test; P-value = 0.11). Of the SNPs occurring within genes, 11 are nonsynonymous changes (nsSNP), two are synonymous (sSNP), and one is a nonsense mutation. All but one of the 11 nsSNPs causes a non-conservative amino acid change. The number of SNPs observed between these two strains is too small to determine whether they were caused by random events, but they are distributed evenly across the genome. Furthermore, the ratio of nsSNPs versus sSNPs, as well as their distribution across many functional gene categories, is consistent with other published reports of inter-strain *Y. pestis* SNP patterns [16].

**Table 1:** 19 SNPs discovered in the comparison of the CO92 and FV-1 genome sequences.

ID	CO92 Genome Position	CO92/FV-1	Within Gene	Substitution	Amino Acid Change CO92/FV-1	CO92 Gene ID (gene name)	Description
a	315608	C/T	Yes	Nonsynonymous	R/Q	YPO0309	Putative exported protein
b	2034614	A/G	Yes	Nonsynonymous	D/G	YPO0706 (flhB)	Flagellar biosynthetic protein FlhB
c	2246196	**G/T**	No	–	–	–	–
d	2862941	**G/T**	Yes	Nonsynonymous	R/S	YPO2550 (nuoG)	NADH dehydrogenase I chain G
e	2872402	T/C	No	–	–	–	–
f	4403914	T/C	Yes	Nonsynonymous	S/G	YPO3920	ABC transporter protein
g	138177	T/C	Yes	Synonymous	–	YPO0126 (malQ)	4-alpha-glucanotransferase
h	1448314	C/T	No	–	–	–	–
i	2320415	**G/T**	Yes	Nonsynonymous	G/C	YPO2045	Putative hemolysin
j	3667446	G/A	Yes	Nonsynonymous	E/K	YPO3287	Two-component system response regulator
k	625366	**G/T**	Yes	Nonsense	E/OCH	YPO0580 (uxaB)	Altronate oxidoreductase
l	4428477	C/T	Yes	Nonsynonymous	G/E	YPO3941 (glgX)	Putative alpha-amylase
m	2278317	A/G	Yes	Nonsynonymous	V/A	YPO2005	Putative exported protein
n	1178178	T/C	No	–	–	–	–
o	2968425	A/G	No	–	–	–	–
p	2300659	**T/G**	Yes	Nonsynonymous	D/A	YPO2029	Putative exported protein
q	2619611	**T/G**	Yes	Nonsynonymous	E/A	YPO2328	Putative membrane protein
r	3739401	**C/A**	Yes	Synonymous	–	YPO3352	Putative Zinc-binding dehydrogenase
s	4579183	A/G	Yes	Nonsynonymous	S/G	YPO4060 (fdhD)	Putative formate dehydrogenase

Bold = transversion mutation

### Phylogenetic analysis

All SNPs discovered between CO92 and FV-1 were screened across a collection of DNA from 24 *Yerinia* isolates (Table 2). The screening set included 20 diverse *Y. pestis* isolates from North America, as well as the two discovery strains (FV-1 and CO92), which were included as positive controls. Two additional isolates were included to provide an outgroup for rooting the phylogenetic tree: one *Y. pseudotuberculosis* (nearest-neighbor) isolate from France and one *Y. pestis* isolate from Kenya. Unique SNP genotypes were identified among the screened samples and one representative of each genotype was included in a neighbor-joining analysis. The subsequent phylogenetic hypothesis was rooted on the genotype assigned to the *Y. pseudotuberculosis* isolate.

**Table 2:** Twenty-four *Yersinia* spp. strains used to screen FV-1/CO92 SNPs.

Strain ID	Source^a^	Species (group)^b^	Country	State	County	Genotype^c^
32953	IP	*Y. pseudotuberculosis*	France	–	–	0
Kenya	Dugway	*Y. pestis* (1.ANT)	Kenya	–	–	0
94A-0226	CDH	*Y. pestis* (1.ORI)	USA	CA	Inyo	0
90A-3290	CDH	*Y. pestis* (1.ORI)	USA	CA	Kern	0
87A-5209	CDH	*Y. pestis* (1.ORI)	USA	CA	Monterey	0
Shasta	USAMRIID	*Y. pestis* (1.ORI)	USA	CA	Shasta	0
85A-4160	CDH	*Y. pestis* (1.ORI)	USA	CA	Siskiyou	0
86A-5151	CDH	*Y. pestis* (1.ORI)	USA	CA	Trinity	0
86A-2907	CDH	*Y. pestis* (1.ORI)	USA	CA	Ventura	0
86A-5331	CDH	*Y. pestis* (1.ORI)	USA	CA	Butte	1.1
94A4772	CDH	*Y. pestis* (1.ORI)	USA	CA	El Dorado	1.1
87A-4884	CDH	*Y. pestis* (1.ORI)	USA	CA	Nevada	1.1
90A-5414	CDH	*Y. pestis* (1.ORI)	USA	CA	Placer	1.1
5190-A-79	CDH	*Y. pestis* (1.ORI)	USA	CA	LA	1.2
91A-3263	CDH	*Y. pestis* (1.ORI)	USA	CA	Riverside	1.2
83A-3817	CDH	*Y. pestis* (1.ORI)	USA	CA	San Bernardino	1.2
94A-8821	CDH	*Y. pestis* (1.ORI)	USA	CA	San Diego	1.2
99A-6797	CDH	*Y. pestis* (1.ORI)	USA	CA	Sierra	1.2
92A-5272	CDH	*Y. pestis* (1.ORI)	USA	CA	Tulare	1.2
1171	USAMRIID	*Y. pestis* (1.ORI)	USA	UT	Juab	2.1
242	USAMRIID	*Y. pestis* (1.ORI)	USA	CO	Denver	2.3
South Park	USAMRIID	*Y. pestis* (1.ORI)	USA	CO	Park	2.3
CO92	ADHS	*Y. pestis* (1.ORI)	USA	CO	Chaffee	CO92
FV-1	NAU	*Y. pestis* (1.ORI)	USA	AZ	Coconino	FV-1

a IP, Institute Pasteur; Dugway, Dugway Proving Grounds; CDH, California Department of Health Services; USAMRIID, United States Army Medical Research Institute of Infectious Diseases; ADHS, Arizona Department of Health Services; NAU, Northern Arizona University.

b Molecular groups of *Y. pestis* based upon Achtman *et al.* [5]

c See Table 3 and Figure 1.

Including the two discovery strains, seven SNP genotypes were identified among the 24 screening isolates (Table 3). As predicted by previous studies [17], phylogenetic analysis of the seven genotypes yielded a linear phylogeny (Figure 1). A linear phylogeny occurs due to discovery bias and a lack of any reversal mutations in the samples studied. It is important to note that, excluding the two discovery strains, only 20 North American *Y. pestis* isolates were screened across the SNPs and these isolates are not a complete representation of all of the geographic locations where plague occurs in North America. It seems quite plausible that additional SNP genotypes will be discovered when these SNPs are screened across a larger isolate collection (currently unavailable), though these should still be consistent with a linear phylogenetic topology.

**Figure 1:**
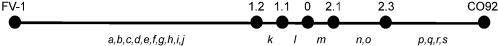
Phylogenetic relationships among the seven *Y. pestis* SNP genotypes identified in this study. Neighbor-joining tree was created using the data presented in Table 3 and MEGA2 software [23], and was rooted on genotype 0, as this genotype was assigned to the two outgroup isolates (see text). The position of the 19 SNPs (Table 1) are indicated below the tree. Individual genotypes or nodes were named based upon their position relative to genotype 0.

**Table 3:** Seven SNP genotypes identified in this study.

Genotype^a^	SNP^b^																			Isolates^c^
	a	b	c	d	e	f	g	h	i	j	k	l	m	n	o	q	p	r	s	
FV-1	T	G	T	T	C	C	C	T	T	A	T	T	G	C	G	G	G	A	G	1
1.2	C	A	G	G	T	T	T	C	G	G	T	T	G	C	G	G	G	A	G	6
1.1	C	A	G	G	T	T	T	C	G	G	G	T	G	C	G	G	G	A	G	4
0	C	A	G	G	T	T	T	C	G	G	G	C	G	C	G	G	G	A	G	9
2.1	C	A	G	G	T	T	T	C	G	G	G	C	A	C	G	G	G	A	G	1
2.3	C	A	G	G	T	T	T	C	G	G	G	C	A	T	A	G	G	A	G	2
CO92	C	A	G	G	T	T	T	C	G	G	G	C	A	T	A	T	T	C	A	1

a Genotypes are named based upon their phylogenetic position (Figure 1).

b See Table 1.

c Number of screening isolates assigned to each genotype (24 total; see text).

Some of the SNPs at the ends of the tree may be specific to the discovery strains (e.g., SNPs a–j may be specific to FV-1 and SNPs p–s may be specific to CO92). However, besides CO92, only two additional isolates from Colorado were included in the screening set and no additional Arizona isolates were included in the set. To determine if these SNPs are indeed specific to the discovery strains, it will be necessary to query these SNPs against isolates that are very closely related to the discovery strains.

## Materials and Methods

### Genome Sequencing

The *Y. pestis* FV-1 isolate used in this study was obtained from a plague epizootic in a Gunnison's prairie dog (*Cynomys gunnisoni*) colony near the city of Flagstaff, Arizona, U.S.A. in 2001 [12]. Approximately10 μg of *Y. pestis* strain FV-1 genomic DNA was randomly sheared using a GeneMachines Hydroshear instrument [18]. The sheared BAC-derived fragments were subjected to end-repair with Klenow and T4 DNA polymerase, ligated to BstXI linkers, and size selected on a 1% low-melt agarose gel. Linker-ligated DNA fragments in the size range of 3–4 kb were purified and ligated to BstXI-digested pOTWI3 vector (provided by the Whitehead Institute/MIT Center for Genome Research). The ligated DNA was electroporated into T1-phage-resistant DH10B *E. coli* cells (Invitrogen), and the resulting transformed cells were plated onto LB agar plates containing 25 μg/ml chloramphenicol. A total of 33,600 random plasmid subclones were selected and DNA was purified for sequencing using the Concert96 plasmid purification kit (Invitrogen) or by solid-phase reversible immobilization (SPRI) magnetic beads (SprintPrep; Agencourt Bioscience Corporation). Sequencing reactions were performed from both ends of plasmid inserts using BigDye Terminator/Version 3.1 (Applied Biosystems) and universal M13 primers (forward and reverse) for 35 cycles in Perkin Elmer 9700 thermocycyclers. Raw sequence data was produced on Applied Biosystems 3730xl sequencing instruments and analyzed with the Phred basecaller software [19]. A total of 62,649 reads were assembled using the whole-genome assemblers Arachne 2.0 [14]. This Whole Genome Shotgun project has been deposited at DDBJ/EMBL/GenBank under the project accession AAUB00000000. The version described in this paper is AAUB01000000.

Dotplot analysis was performed using the program PipMaker using the “single coverage” and “ordered and oriented” options [20].

### Identification and analysis of single-nucleotide polymorphisms (SNPs)

The gapped genome sequence of *Y. pestis* strain FV-1 was compared to the complete genome sequence of strain CO92 (GenBank no. NC_003143) using the program MUMmer [15]. Putative SNPs were identified from this alignment by extracting single-base-pair mismatches. We enforced the criteria that the SNP nucleotide in the FV-1 sequence possess a quality score of Phred-30 or above (sequence quality scores were not available for strain CO92) [21]. We further required that 250 base pairs on either side of the putative SNP in the FV-1 sequence be present (to avoid SNPs at the ends of contigs where sequence quality tends to be poor). A final validation step verified that the SNP was from a unique region of the reference sequence using the program REPuter [22]. Those SNPs located within repetitive sequence were excluded from further analysis.

Detection of SNPs was performed using the Homogenous MassEXTEND (hME) assay as per manufacturers instructions (Sequenom, San Diego CA). Briefly, multiplexed assays were designed using the Sequenom Assay Design 2.0 software. PCR amplification of genomic DNA was performed using the Sequenom massEXTEND 12-plex multiplexing protocol (Sequenom application note 8876-001, R01, CO 040038) for 45 cycles in Perkin Elmer 9700 thermocyclers. The hME primers for each multiplex reaction were spotted onto SpectroCHIPs using the Sequenom CCU RoboDesign nanodipenser, detected on the Sequenom MALDI-TOF mass spectrophotometer (MS), and adjusted to normalize MALDI-TOF MS peak height intensities for subsequent multiplexing reactions. Shrimp alkaline phosphatase (SAP)-treated PCR products were multiplexed using normalized primer concentrations and amplified for 99 cycles in PE 9700 thermocyclers, followed by nanodispensing and MALDI-TOF MS analysis.
